# Initial surgical results of subcostal uniportal robotic lung resection: a study of the perioperative outcomes and a CUSUM analysis of the learning curve

**DOI:** 10.1007/s00595-026-03263-7

**Published:** 2026-08-29

**Authors:** Teruhisa Kawaguchi, Fumiaki Watanabe, Shinji Kaneda, Daisuke Ito, Koji Kawaguchi

**Affiliations:** Department of Thoracic Surgery, Mie University, 2-174, Edobashi, Tsu, 514-8507 Mie Japan

**Keywords:** Single-port robotic surgery, Robotic-assisted thoracoscopic surgery, Learning curve

## Abstract

**Purpose:**

Anatomical lung resection using the da Vinci SP system was recently introduced in Japan. We retrospectively evaluated our initial experience and analyzed the learning curve for the first 53 consecutive cases.

**Methods:**

Fifty-three patients with primary lung cancer underwent anatomical resection, including lobectomy and segmentectomy, using the da Vinci SP system. We reviewed the perioperative outcomes, including the operative time, console time, conversion rates, and complications. The learning curves for operative and console times were assessed using a cumulative summation analysis.

**Results:**

The mean patient age was 71.5 years. Thirty-five and 18 patients underwent lobectomy and segmentectomy, respectively. The mean operative and console times were 207.1 min and 147.5 min, respectively. Two patients (4%) required conversion to an open thoracotomy. Two patients (4%) experienced Clavien-Dindo grade ≥ II postoperative complications, with no severe morbidity. A cumulative summation analysis indicated that the learning curve for lobectomy plateaued after the 14th case, whereas proficiency for all resections was achieved after 18–19 cases.

**Conclusion:**

Anatomical lung resection using the da Vinci SP system can be safely performed with favorable short-term outcomes. Surgeons with experience in multiport robotics may achieve proficiency in approximately 14–18 cases.

**Supplementary Information:**

The online version contains supplementary material available at https://doi.org/10.1007/s00595-026-03263-7.

## Introduction

Minimally invasive approaches in thoracic surgery began with video-assisted thoracoscopic surgery (VATS) in the 1990s [1]. Currently, multiport robot-assisted thoracic surgery (RATS) and uniportal VATS are established approaches [2]. In particular, multiport RATS have been introduced nationwide, with reports confirming their safety [3, 4]. To reduce surgical trauma, single-port approaches using multi-arm robotic platforms have been developed; however, instrument collisions and limited access remain technical challenges [5]. 

The da Vinci SP system (Intuitive Surgical, Inc.), approved for use in Japan in September 2022, enables robotic surgery through a single port with a single arm. As the da Vinci SP system cannula has a diameter of 2.5 cm, the intercostal approach is not recommended. Consequently, a subcostal approach was adopted. Our institution began performing anatomical lung resection via the subcostal approach using the da Vinci SP system in February 2024 [6]. In this study, we retrospectively review the short-term outcomes of our first 53 cases to evaluate the safety and feasibility of this procedure and to provide a detailed analysis of the learning curve.

## Materials and methods

All surgeries and data collection were performed at Mie University Hospital, Tsu, Japan. Clinical outcome data were obtained from hospital records, which were reviewed and approved by the Clinical Research Ethics Review Committee of Mie University Hospital (approval number: H2025-092; approval date: June 3, 2025). All methods were performed in accordance with the relevant guidelines and regulations.

### Patients

We included 53 consecutive patients who underwent anatomical lung resection for primary lung cancer using the da Vinci SP system between February 2024 and July 2025. The inclusion criteria were clinical stage 0 to IV lung cancer without bulky lymph node metastasis or requirement for complex procedures, such as bronchoplasty or pneumonectomy. All surgeries were performed by a single surgeon with 28 years of clinical experience, who had previously performed more than 500 thoracic surgeries using multiport robots. Informed consent was obtained from the participants using the opt-out method.

### Surgical technique using the da Vinci SP

All the procedures were performed using a subcostal approach. Patients were placed in the lateral decubitus position, and the operating table was tilted into a 10–20 deg “mountain fold” position. A 4 cm skin incision was made along the subcostal arch, approximately one fingerbreadth ventral to the eighth rib. The external and internal oblique muscles were incised sequentially to approach the peritoneum. The peritoneum was retracted caudally to apply tension to the diaphragm and partially incised to access the thoracic cavity (Video 1). An access port for the da Vinci SP system was inserted, and CO₂ insufflation was initiated at 8 mmHg using the AirSeal system (CONMED Corporation, Largo, FL, USA). The technique described above was used for both the left- and right-sided procedures.

**Fig. 1:**
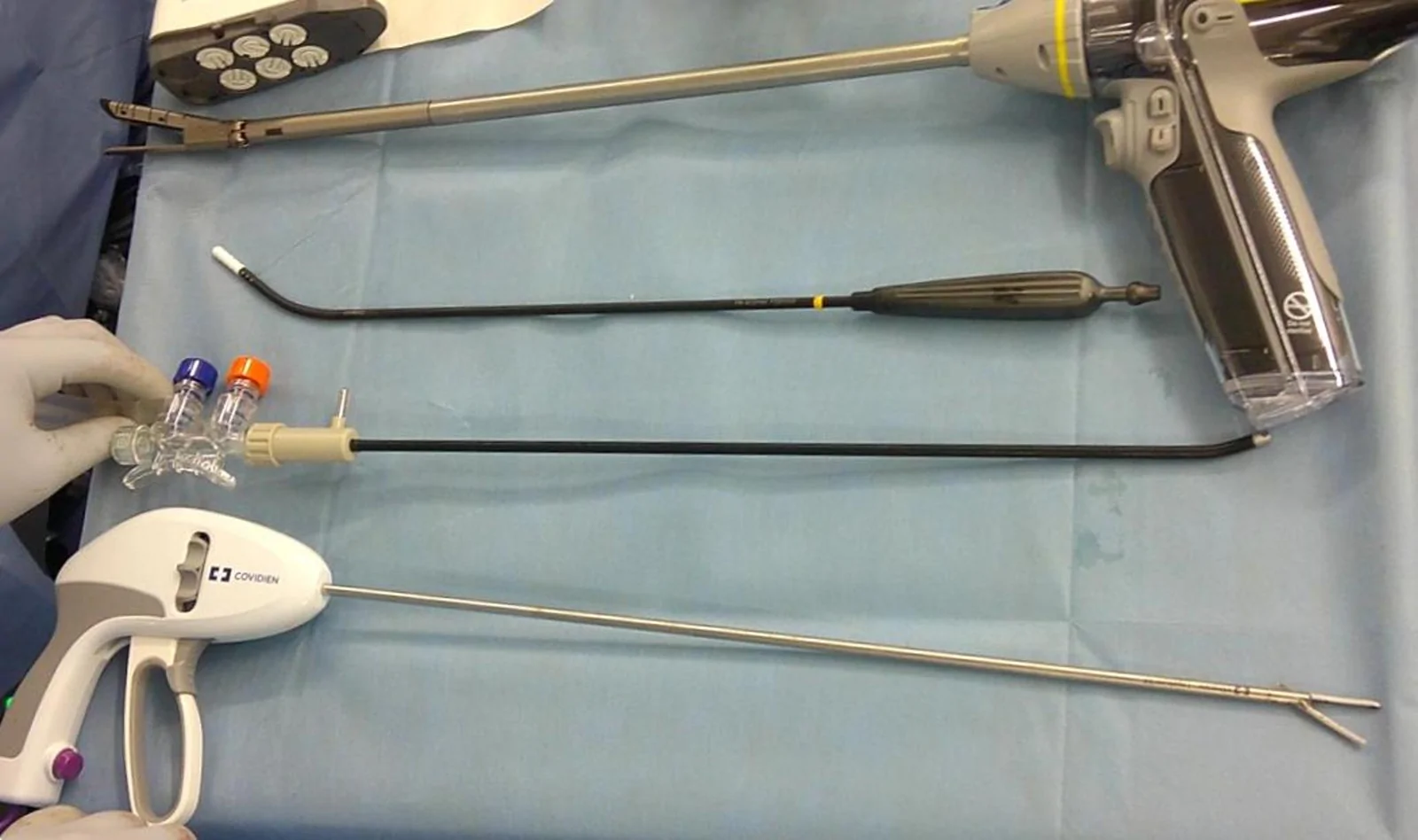
The curved instruments were used by an assistant. From top to bottom of the figure: stapler, curved cotton holder, suction device, and energy device

A round tooth tractor, Cadiere forceps, and Maryland bipolar forceps (Intuitive Surgical, Sunnyvale, CA, USA) were used. The robotic arm was positioned to secure the working space ventral to the port, allowing the assistant to operate in the thoracic cavity. Curved instruments were used to reduce interference with the robotic arm (Fig. 1). A 44-cm-long LigaSure™ (Medtronic plc, Minneapolis, MN, USA) was used as the energy device. Suction was performed by an assistant using a curved suction device with irrigation capability. Vessels ≤ 7 mm in diameter were ligated with silk sutures and divided using an energy device, while vessels > 7 mm in size were divided using a stapler [7]. We selected the extra-long Signia™ (Medtronic plc) as the stapler because it allows angle adjustment within the thoracic cavity, and the anvil moves appropriately. The ventral robotic arm was temporarily removed for stapler insertion.

A 28-Fr thoracic catheter was inserted through the same incision in all cases. The chest was closed by suturing the diaphragm to the muscle layer beneath the costal arch, followed by subcutaneous closure using 3 –0 V-Loc™ (Medtronic plc).

### Postoperative management

All patients were extubated in the operating room. Ambulation and oral intake were resumed on postoperative day (POD) 1. Chest tubes were managed with continuous suction at 5–10 cmH₂O until the following morning and then placed on a water seal. The drain was removed after confirming the absence of air leaks or chylothorax and when the daily drainage volume was less than 200 mL. The patients were discharged after confirming no abnormalities on chest radiography on the day following drain removal.

### Statistical analyses

The primary endpoint was the short-term perioperative outcomes in all 53 patients. The secondary endpoint was the analysis of the learning curve for operative and console times, both for the entire cohort and specifically for lobectomy cases. The learning curves were analyzed using the cumulative summation (CUSUM) method. CUSUM was calculated as CUSUM = ∑^N^_i=1_ (ti – tm), where ti is the operative (or console) time for a given case and tm is defined as the mean operative (or console) time for all cases. The inflection point of the CUSUM curve, which represents the point at which the learning curve was overcome, was identified as the number of cases corresponding to the peak of the curve. This analysis was performed for the entire cohort of 53 anatomical lung resections and a subgroup of 33 lobectomy cases, excluding patients with cN1 disease or a history of induction therapy. All statistical analyses were performed using EZR (Saitama Medical Center, Jichi Medical University, Saitama, Japan), a graphical user interface for R (R Foundation for Statistical Computing, Vienna, Austria).

## Results

The cohort included 53 patients with a mean age of 71.5 years; 27 were male and 26 were female (Table 1). All but one patient had a Performance Status of 0. Forty-three patients had comorbidities, of which 7 had respiratory-related comorbid conditions. The most common clinical stage was Stage I (42 cases, 79%), and only one patient had cN1 disease. One patient received neoadjuvant pembrolizumab therapy.

**Table 1:** Baseline characteristics of the patients. The clinical TNM stage was classified according to the AJCC, 9th edition. IQR: interquartile range

Variable	Median (IQR) / Number (percent)
Age (years)	73 (67-79)
Sex (Men/Women)	27 (51%) / 26 (49%)
Body mass index (kg/m^2^)	22.7 (20.4-23.9)
Brinkman Index	87 (0-860)
*Preoperative ECOG performance status*	
0	52 (98%)
1	1 (2%)
*Preoperative pulmonary function test*	
%VC (%)	99.5 (93.9-107.7)
FEV_1_% (%)	78.4 (73.6-81.6)
Comorbidities	43 (81%)
Respiratory	7 (13%)
Cardiovascular	29 (55%)
Diabetes mellitus	8 (15%)
Other Tumor	21 (40%)
Tumor size (cm)	2.4 (1.5-3.0)
Solid size (cm)	1.7 (1.0-2.6)
*Clinical TNM*	
Tis	3 (6%)
T1	42 (79%)
T2	4 (8%)
T3	4 (8%)
N0	52 (98%)
N1	1 (2%)
*Clinical stage*	
Stage I	44 (83%)
Stage II	6 (11%)
Stage IV	1 (2%)
Neoadjuvant therapy	1 (2%)

The procedure included 35 lobectomies and 18 segmentectomies (Table 2). The mean operative time was 207.1 min, the mean console time was 147.5 min, and the mean blood loss was 51.1 ml. An additional port was required in three cases (6%): one for dissection of the A3 branch in the left upper lobectomy (17th case) and two for lysis of apical adhesions (20th and 34th cases). Conversion to thoracotomy occurred in two cases (4%): one was due to lymph node infiltration into the pulmonary artery (8th case: neoadjuvant case) and the other was due to bleeding from the root of A6 during dissection (30th case).

**Table 2:** Intraoperative and postoperative variables. Pathological stage was classified according to the AJCC, 9th edition. IQR: interquartile range

Variable	Median (IQR) / Number (percent)
*Lobectomy*	35 (66%)
Right upper lobe	13
Right middle lobe	3
Right lower lobe	6
Left upper lobe	5
Left lower lobe	8
*Segmentectomy*	18 (34%)
Right S1	1
Right S2	1
Right S1+2	1
Right S6	4
Right S7+10	1
Right S9+10	1
Right S10	2
Left S1+2	1
Left S1+2+3	1
Left S4+5	1
Left S6	3
Left S8	1
*Nodal Dissection*	
ND0	4
ND1	18
ND2	31
Operative time (min)	201 (176-228)
Console time (min)	142 (121-170)
Bleeding (ml)	10 (10-10)
Total number of staples	7 (6-8)
Transfusion	1 (2%)
Conversion to thoracotomy	2 (4%)
Additional port needed	3 (6%)
Total number of lymph nodes harvested	8 (3-13)
*Histological type*	
Adenocarinoma	46 (87%)
Squamous cell carcinoma	6 (11%)
Adenosquamous carcinoma	1 (2%)
*Pathological stage*	
0	5 (9%)
I	40 (75%)
II	3 (6%)
III	3 (6%)
IV	2 (4%)
Chest tube duration (days)	2 (2-3)
Postoperative hospital stays (days)	5 (5-6)
*Postoperative complications within 30 days (Clavien *– *Dindo classification)*	
None	51 (96%)
II	2 (4%)
Pneumonia	1
Reflux esophagitis	1

The most common histological type was adenocarcinoma (*N* = 46). The mean duration of postoperative chest tube drainage was 2.6 days, and the mean postoperative hospital stay was 5.9 days. Clavien-Dindo grade ≥ II postoperative complications occurred in two patients (4%), including one case of pneumonia and one case of reflux esophagitis.

**Fig. 2:**
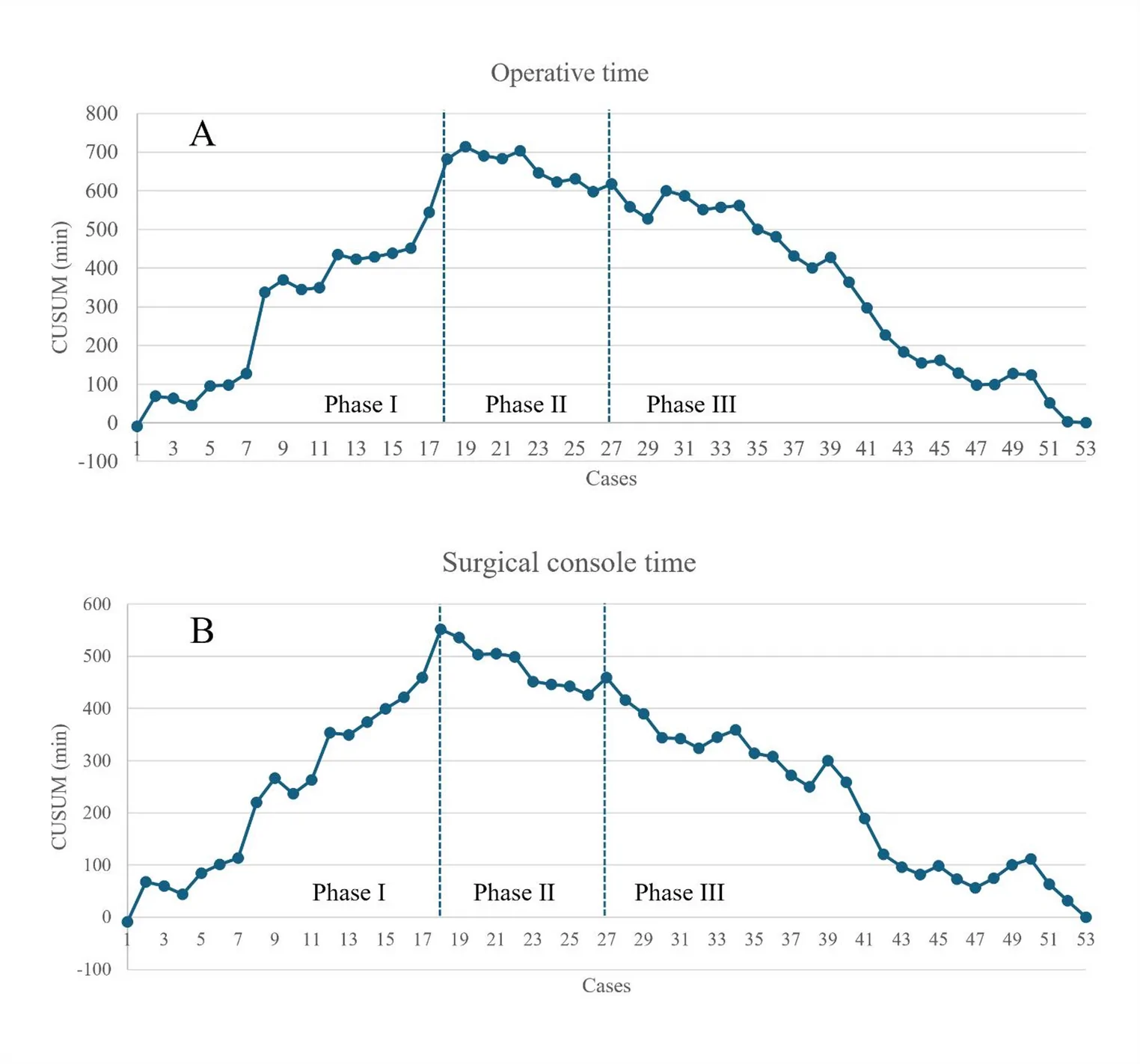
The CUSUM plot. (**a**) The CUSUM analysis of the operative time for all 53 anatomical resections. The peak of the curve was at the 19th case, indicating the inflection point of the learning curve. (**b**) The CUSUM analysis of the surgical console time for all 53 anatomical resections. The peak of the curve was at the 18th case

**Fig. 3:**
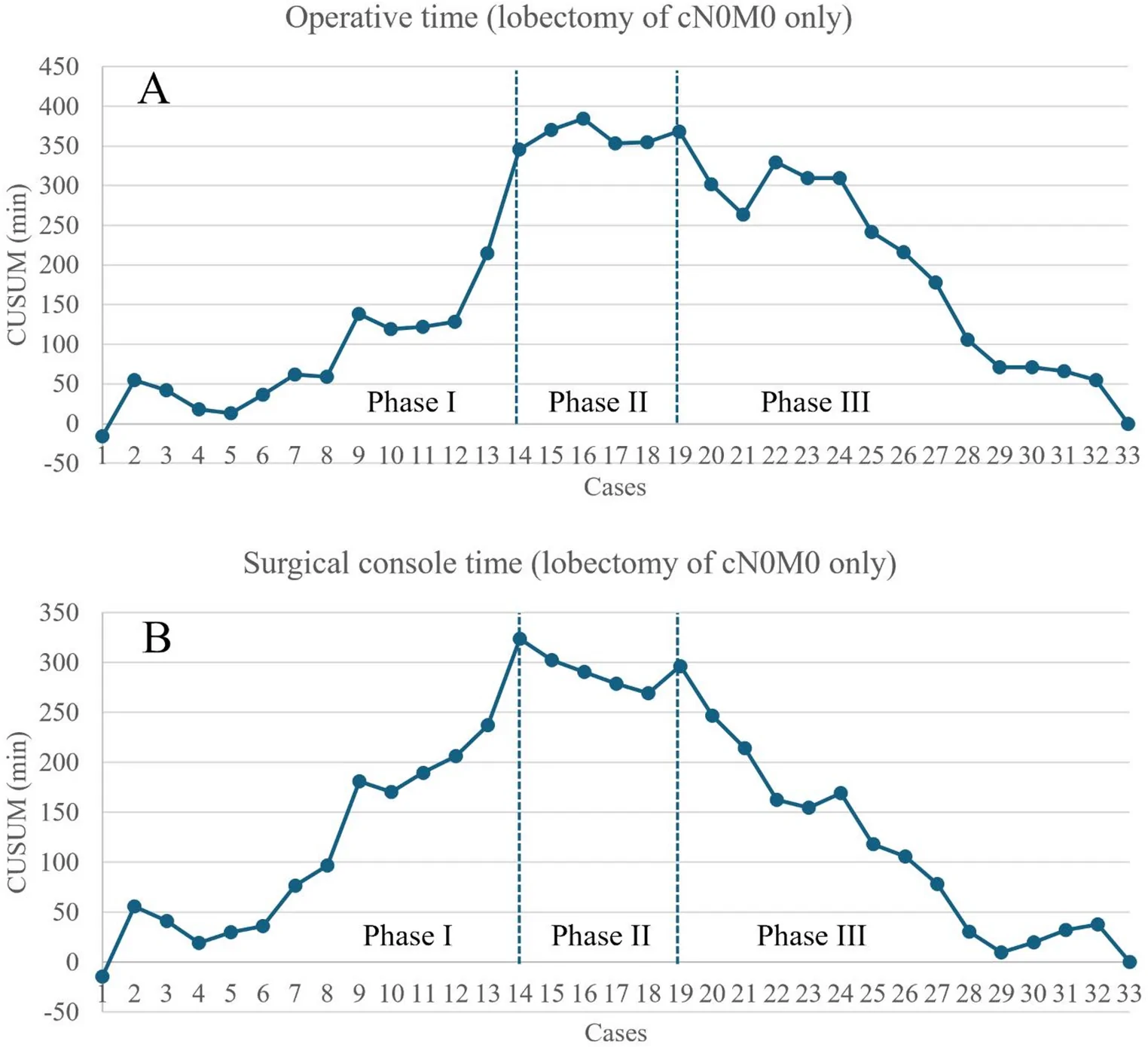
The CUSUM plot for lobectomy excluding patients with cN1 disease or a history of induction therapy. (**a**) The CUSUM analysis of operative time for 33 cases. The peak of the curve was at the 14th case. (**b**) The CUSUM analysis of the surgical console time for 33 lobectomy cases. The peak of the curve was at the 14th case

A CUSUM analysis for all 53 anatomical resection cases indicated a peak of the operative-time curve in the 19th case (Fig. 2a) and a peak of the console-time curve in the 18th case (Fig. 2b). We defined Phase I of the learning curve for operative time and surgical console as cases 1–18, Phase II as cases 19–27, and Phase III as cases 28 and beyond. A CUSUM analysis demonstrated a faster learning curve for a specific subgroup of 33 lobectomy cases. The inflection point for operative time was reached in the 14th case (Fig. 3a) and the inflection point for surgical console time was reached in the 14th case (Fig. 3b). We defined Phase I of the learning curve for the operative time and surgical console time in lobectomies as cases 1–14, Phase II as cases 15–19, and Phase III as cases 20 and beyond.

## Discussion

We report one of the first Japanese single-center series describing the initial clinical outcomes of 53 anatomical lung resections performed via a subcostal approach using the da Vinci SP system. Our results indicate that the procedure can be safely introduced, with low conversion and complication rates and a relatively rapid learning curve for an experienced robotic surgeon.

Recent reports, including a retrospective series from Korea and a prospective multicenter feasibility study from the United States, have similarly suggested that single-port subcostal robotic major pulmonary resection is feasible and safe [8, 9]. A US study reported a 0% conversion rate to multiport RATS or open surgery in 19 lobectomy cases across six centers. Similarly, a Korean study of 25 patients reported no conversion to thoracotomy, with only one case (4%) requiring an additional port for adhesions. In our series, the conversion rate to thoracotomy was 4% (2 cases), and an additional port was required in 6% of the cases (3 cases). Although we experienced some conversions due to bleeding, the subcostal approach offers the advantage of a free lateral thoracic space, which allows the assistant to operate without interference from the main robotic body, thus enabling controlled conversion in both instances. We also found that lysis of dense apical adhesions can be challenging owing to the limitations of the da Vinci SP arm, a finding consistent with that reported by Lee et al. Despite some potential selection bias toward less advanced cases in our initial experience, these results strongly support that anatomical lung resection with the da Vinci SP system is feasible with a high completion rate when performed by an experienced robotic surgeon.

A key finding of our study was the rapid learning curve of an experienced robotic surgeon transitioning to the da Vinci SP system. Our CUSUM analysis accurately identified the inflection point in RATS proficiency using the da Vinci SP system in approximately 18 cases. This finding is highly consistent with the extensive literature on the learning curve for conventional multiport RATS. A large systematic review concluded that technical proficiency for RATS lobectomy is typically achieved after approximately 25 cases [10]. Single-institution studies using CUSUM analyses have similarly identified this inflection point at approximately 28–32 cases [11, 12]. Our results align with emerging data on the learning curve for uniportal RATS. For instance, Mercadante et al., in an early single-center series of 24 uniportal RATS lobectomy cases, noted a “very fast learning curve” with operative times decreasing significantly between the first 10 and the last 10 cases [13]. Another analysis by Wu et al. estimated that approximately 20 cases were needed to achieve technical proficiency in uniportal RATS [14]. It should also be noted that uniportal RATS has been introduced with acceptable safety in early Japanese experiences [15]. The rapid progression observed in our study may be attributed to the surgeon’s extensive prior experience with the multiport da Vinci Xi system. This is consistent with the previous findings. For instance, one report suggested that an individual surgeon already proficient in VATS could adapt to RATS in five to 5–6 cases, even as the team continued to learn [12]. This supports the finding that surgeons already proficient in other minimally invasive techniques adapt more quickly to new robotic platforms. Therefore, our finding of the learning curve plateau after an initial phase of approximately 14–18 cases is not only internally valid, but also well supported by benchmarks in both the relevant literature on multiport and uniportal RATS.

Interpretation of the postoperative complication rates must consider the differences in the study design. Compared with our study (4% grade ≥ II, 0% grade ≥ III) and the Korean report (4% Grade IIIa), the US study reported a higher rate of Grade III complications (21.1%). However, the authors of the US study attributed this to two factors: first, as a prospective clinical trial, minor adverse events, such as fatigue and pain, were more rigorously documented than in retrospective studies; second, two of the four major complications occurred in a single complex case that required chest wall resection. Considering this context, they concluded that the complication profile was within the reported range for other RATS and uniportal approaches. Indeed, multiple reviews and cohort studies have affirmed that uniportal RATS is a safe and feasible technique when performed by experienced surgeons, with low conversion rates and complication profiles comparable to those of standard approaches [16, 17]. 

A common technical challenge across all three studies was the lack of an integrated robotic stapler for the da Vinci SP system, necessitating manual stapling by an assistant. This underscores the critical importance of a skilled assistant proficient in the procedure. To minimize instrument interference, we used long-handled curved instruments, similar to those used in uniportal VATS.

Finally, the subcostal approach using the da Vinci SP system may reduce intercostal nerve irritation and postoperative pain. However, pain-related outcomes were not evaluated in this study. The US study did not collect standardized pain scores, thus highlighting this as a current knowledge gap. This should be assessed in future prospective multicenter studies using standardized pain scores.

The limitations of our study include its retrospective, single-center, single-surgeon design and the relatively small sample size. Given the background of this dataset, a comparison of the learning curves with other systems or approaches was considered difficult and was not performed. Second, during the same period, we performed 22 mediastinal tumor surgeries, indicating that this was not a pure learning curve for the da Vinci SP system [18]. However, since mediastinal tumor surgery involves straightforward techniques, this review focuses exclusively on lung resection procedures. This study provides a more objective learning curve assessment than simple time-series comparisons by employing robust CUSUM analysis. Caution is warranted when extrapolating favorable outcomes to a broader patient population or less experienced surgeons.

In conclusion, our initial experience demonstrated that anatomical pulmonary resection via a subcostal approach using the da Vinci SP system is a safe and feasible procedure, with acceptable perioperative outcomes. For an experienced robotic surgeon, the learning curve is manageable and rapid, with proficiency achieved after approximately 14–18 cases as demonstrated by a CUSUM analysis. Large-scale comparative trials are required to fully elucidate the definitive role and potential benefits of this technique in thoracic surgery.

## Electronic Supplementary Material

Below is the link to the electronic supplementary material.

Supplementary Material 1
